# A single amino acid change in histone H4 enhances UV survival and DNA repair in yeast

**DOI:** 10.1093/nar/gks924

**Published:** 2012-10-22

**Authors:** Ronita Nag, Feng Gong, Deirdre Fahy, Michael J. Smerdon

*Nucleic Acids Res.* 2008; **36**, 3857–3866. doi:10.1093/nar/gkn311

The authors would like to apologize for an error in Figure 6B (showing NER of *RPB2* locus and *HML* locus in *RAD26* deleted mutants) of this article. The index in the above-mentioned figure incorrectly appears as:


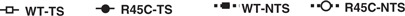


The index should have appeared as shown in the figure below:


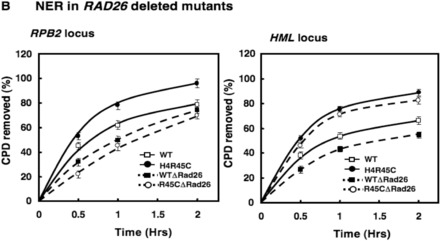


This correction does not influence the validity of the results and conclusions of this article.

